# White matter microstructural properties in bipolar disorder in relationship to the spatial distribution of lithium in the brain

**DOI:** 10.1016/j.jad.2019.04.075

**Published:** 2019-06-15

**Authors:** Joe Necus, Nishant Sinha, Fiona Elizabeth Smith, Peter Edward Thelwall, Carly Jay Flowers, Peter Neal Taylor, Andrew Matthew Blamire, David Andrew Cousins, Yujiang Wang

**Affiliations:** a Institute of Neuroscience, Newcastle University, Newcastle upon Tyne, NE1 7RU, United Kingdom; b Interdisciplinary Computing and Complex BioSystems (ICOS), School of Computing Science, Newcastle University, Newcastle upon Tyne, NE1 5TG, United Kingdom; c Institute of Cellular Medicine, Newcastle University, Newcastle upon Tyne, NE1 7RU, United Kingdom; d Newcastle Magnetic Resonance Centre, Newcastle University, Campus for Ageing and Vitality, Newcastle upon Tyne, NE4 5PL, United Kingdom; e Northumberland Tyne and Wear NHS Foundation Trust, Newcastle upon Tyne, NE3 3XT, United Kingdom; f Institute of Neurology, University College London, London, WC1N 3BG, United Kingdom

**Keywords:** Bipolar disorder, Lithium, Magnetic resonance imaging, Diffusion imaging, White matter, Fractional anisotropy

## Abstract

**Background:**

Lithium treatment is associated with an increase in magnetic resonance imaging derived measures of white matter integrity, but the relationship between the spatial distribution of brain lithium and white matter integrity is unknown.

**Methods:**

Euthymic patients with bipolar disorder receiving lithium (*n* = 12) and those on other medications but naïve to lithium (*n* = 17) underwent diffusion imaging alongside matched healthy controls (*n* = 16). Generalised fractional anisotropy (gFA) within white matter was compared between groups using a standard space white matter atlas. Lithium-treated patients underwent novel multinuclear lithium magnetic resonance imaging (^7^Li-MRI) to determine the relative lithium concentration across the brain. The relationship between ^7^Li-MRI signal intensity and gFA was investigated at the resolution of the ^7^Li-MRI sequence in native space.

**Results:**

Lithium-treated bipolar disorder and healthy control groups had higher mean white matter gFA than the bipolar disorder group treated with other medications (*t* = 2.5, *p* < 0.05; *t* = 2.7, *p* < 0.03, respectively). No differences in gFA were found between patients taking lithium and healthy controls (*t* = 0.02, *p* = 1). These effects were seen consistently across most regions in the white matter atlas. In the lithium-treated group, a significant effect of the ^7^Li-MRI signal in predicting the gFA (*p* < 0.01) was identified in voxels containing over 50% white matter.

**Limitations:**

Cross-sectional evaluation of a relatively small cohort.

**Conclusions:**

The higher gFA values observed in the lithium-treated bipolar disorder group suggests that long-term lithium is associated with greater white matter integrity. Our novel analysis supports this further, showing a positive association between white matter gFA and the spatial distribution of lithium.

## Highlights

• Using a novel analysis, we combined diffusion tensor imaging and ^7^Li magnetic resonance imaging to investigate the effects of lithium in bipolar disorder (BD).
• White matter gFA values were higher in those taking lithium compared to other medications, suggesting that lithium is associated with greater white matter integrity.
• We report a positive association between white matter gFA and the spatial distribution of lithium in the brain.

## 1 Introduction

Bipolar disorder is a major mental illness affecting 1% of the world's population. Although numerous treatments are available, lithium retains a key position in major treatment guidelines, notably for the prevention of manic episodes (Severus et al., 2014). The mechanisms by which lithium achieves its effects are not fully understood and we lack detailed information about its tissue level distribution and how this relates to its actions in the brain.

Various neuroimaging techniques have been used to identify the structural and functional changes that occur in the brain during treatment with lithium. In bipolar disorder, cross-sectional structural magnetic resonance imaging (MRI) studies have revealed that treatment with lithium is associated with increased grey matter volume and cortical thickness (Hafeman et al., 2012; Hibar et al., 2018), and longitudinal studies have reported progressive increases in measures of grey matter volume following lithium initiation (Lyoo et al., 2010). These studies have generally shown that the effects of lithium on MRI estimates of grey matter volume are not uniform across the brain, and may localise to candidate regions such as the hippocampus, amygdala, anterior cingulate and prefrontal cortex (Zung et al., 2016; Hibar et al., 2018; Hibar et al., 2016; Monkul et al., 2007).

Whilst the effects of lithium on white matter are not fully understood, in bipolar disorder it has been consistently shown to positively influence measures of white matter integrity. Diffusion MRI (dMRI) enables the examination of the microstructural properties of tissue by mapping water molecule diffusion. In pure fluid states, water diffuses equally in all directions (isotropic). In neurons, water cannot readily cross the hydrophobic myelin sheaths and diffusion is restricted (anisotropic) along the direction of the axon. The parallel configuration of axons in white matter renders this directional diffusion detectable at the microstructural scale, reflected in the dMRI-derived parameter of fractional anisotropy (FA). Decreases in FA within white matter have been shown to correspond with demyelination and axonal injury (Song et al., 2002), such that FA is held to be a measure of white matter integrity. A number of studies have examined regional FA in bipolar disorder, generally reporting low FA values in white matter regions and tracts involved in mood regulation (Adler et al., 2006; Foley et al., 2018; Bruno et al., 2008; Benedetti et al., 2011). The findings from whole brain dMRI studies are heterogeneous, but meta-analysis has identified two significant clusters of low FA in the right hemisphere (parahippocampal gyrus and the anterior cingulate/subgenual cingulate cortex) (Marlinge et al., 2014). White matter FA is greater in patients with bipolar disorder taking lithium compared to those on other medications, and the differences may be a function of the duration of treatment (Macritchie et al., 2009; Gildengers et al., 2015; Haarman et al., 2016). In a recent region of interest (ROI) study using longitudinal dMRI data from adolescents with bipolar disorder (Kafantaris et al., 2017), baseline FA values in the cingulum hippocampus were lower than healthy controls and increased following treatment with lithium, the effect being most marked in responders. However, in the absence of a whole brain analysis, it remains unclear whether this effect of lithium is regionally specific or global.

Regionally specific effects of lithium would indicate either that brain regions are differentially sensitive to its effects or that the drug itself is heterogeneously distributed across the brain. Recent advances in multinuclear MRI (^7^Li-MRI) have permitted the rapid determination of brain lithium distribution *in vivo* (Smith et al., 2018) and demonstrated a heterogeneous distribution of lithium in bipolar disorder, in keeping with previous rodent imaging and human magnetic resonance spectroscopy studies (Lee et al., 2012; Zanni et al., 2017). It would therefore be reasonable to propose that the effects of lithium on white matter integrity relate to its regional concentration and the combination of ^7^Li-MRI and dMRI affords the capability to explore this.

In this study we compared generalised FA (gFA), a measure analogous to FA but better able to account for crossing fibres (Tuch, 2004), across white matter ROI in patients with bipolar disorder receiving lithium, those taking other maintenance medications but naïve to lithium, and a healthy comparator group. Patients receiving lithium additionally underwent an assessment of brain lithium distribution by ^7^Li-MRI. We hypothesised that gFA values in patients receiving lithium treatment would exceed those of patients on other treatments and be comparable to healthy controls, and that the areas of the brain with the greatest signal intensity on ^7^Li-MRI would be those with the largest magnitude gFA in the lithium treated group.

## 2 Methods

### 2.1 Subjects and assessments

Twenty-nine euthymic subjects with a diagnosis of bipolar disorder (I or II) and sixteen healthy control subjects recruited to the Bipolar Lithium Imaging and Spectroscopy Study (BLISS) were investigated. Of those with bipolar disorder, twelve were taking lithium as a long-term treatment (Bipolar Disorder Lithium, BDL) and seventeen were taking other maintenance treatments but were naïve to lithium (Bipolar Disorder Control, BDC). The healthy control subjects (HC) had no history of psychiatric illness and were not taking any psychotropic medications. Subjects attended a screening visit to confirm eligibility and underwent a structured clinical interview using the NetSCID diagnostic tool (a validated online version of the Structured Clinical Interview for DSM-5 Criteria; Telesage, Inc., Chapel Hill, NC, USA). Interviews and objective ratings were conducted by a trained clinical research assistant (CJF) and discussed with a senior psychiatrist (DAC). All subjects were 18–65 years of age and between 50 and 150 Kg in weight (upper limit determined by MRI scanner bed restrictions). Across all groups, subjects were excluded if they had a contraindication to magnetic resonance examination (including claustrophobia), a current or past medical condition deemed likely to tangibly affect brain structure, a substance use disorder (current or to a significant degree in the past; NetSCID Module E), a weekly alcohol intake exceeding 21 units (self-reported), a learning disability or an impairment of capacity. Patients were excluded if they were currently liable to detention under the Mental Health Act 1983 (amended 2007). Comorbid psychiatric diagnosis in the patients, assessed using the NetSCID, was permissible (excluding neurodevelopmental, substance use as previously described and neurocognitive disorders) so long as the primary diagnosis was bipolar disorder, confirmed by a senior psychiatrist (DAC) reviewing case notes as required. Euthymic mood state was confirmed at entry to the study, defined as scores of less than seven on both the 21-item Hamilton Depression Rating Scale (HAM-D) and the Young Mania Rating Scale (YMRS). BDL subjects were required to have been taking lithium carbonate (Priadel™ modified release once daily) regularly for at least one year at the time of recruitment (target therapeutic range 0.6–1.0 mmol/L) and all were taking at least one concomitant medication. BDL subjects completed the Lithium Side Effects Rating Scale (LISERS), a self-administered scale rating the common side effects of lithium, each on a four-point severity scale and expressed as a summated score (Haddad et al., 1999). All scans were performed at 9 am and the BDL subjects were instructed to take their lithium as usual the night before and submitted to a blood test immediately prior to scanning to measure their serum lithium concentration. All subjects provided written informed consent and the study was granted a favourable ethical opinion by a United Kingdom National Research Ethics Committee (14/NE/1135).

*Group comparison:* Statistical data analysis was performed using Matlab R2016b (Mathworks® Inc., Natick, MA, USA). Prior to comparison of groups, continuous variables were tested for normality of distribution using the Kolmogorv–Smirnov test and by examining Q–Q plots. Normally distributed variables were examined using the Student's *t*-test, non-parametric data were compared using the Mann–Whitney *U* test. Dichotomous data with samples containing less than five per cell were analysed using a Fisher's exact test, whilst other dichotomous data were analysed using a χ^2^ test. All statistical tests were regarded significant at *p* < 0.05.

### 2.2 Image acquisition

*Scanner and coil systems:* All magnetic resonance data were acquired on a Philips 3T Achieva scanner (Philips Medical Systems, Best, The Netherlands) with ^1^H structural and diffusion weighted imaging performed using a Philips 8-channel SENSE head coil in all subjects. ^7^Li-MRI, together with an additional ^1^H structural image for the purposes of registration, was performed using a double tuned ^1^H/^7^Li radiofrequency (RF) birdcage head coil (RAPID Biomedical, Rimpar, Germany).

*T1-weighted imaging acquisition:* 3D T1-weighted images (T1w) of brain anatomy, acquired in all subjects using the 8-channel SENSE head coil, were obtained with a ^1^H gradient echo sequence (TR = 9.6 ms, TE = 4.6 ms, FOV = 240 × 240 × 180 mm^3^, acquisition matrix = 240 × 208 × 180, acquisition voxel size = 1 × 1.15 × 1 mm^3^, reconstructed into a matrix size of 256 × 256 × 180, 1 average). Those in the BDL group were also scanned using a double tuned ^1^H/^7^Li head coil, with a ^1^H gradient echo sequence (TR = 8.2 ms, TE = 4.6 ms, FOV = 216 × 240 × 175 mm^3^, acquisition matrix = 180 × 200 × 146, acquisition voxel = 1.2 × 1.2 × 1.2 mm^3^ reconstructed into a matrix size of 240 × 240 × 146, 1 average). The total acquisition time for each sequence was less than 5 min.

*Diffusion-weighted imaging acquisition*: dMRI data was acquired using a single-shot pulsed gradient spin echo (EPI) sequence (TR = 10,200 ms, TE = 74 ms). For each subject, 64 b1000 diffusion-weighted volumes were collected along non-collinear directions. In addition, one b0 volume was also acquired. Each volume consisted of 55 transverse slices (FOV = 240 × 240 × 137.5 mm^3^, voxel size = 2.5 mm isotropic, no gap). The total acquisition time for this sequence was less than 13 min.

*^7^Li magnetic resonance imaging:* ^7^Li 3D Balanced Steady State Free Precession (bSSFP) gradient echo acquisition (^7^Li-MRI) was acquired in eleven of the BDL subjects using a protocol constructed for maximal ^7^Li signal amplitude (FOV = 480 × 480 × 175 mm^3^, 20 × 19 × 7 acquisition matrix and 24.0 × 25.3 × 25.0 mm^3^ voxel size, with TR = 9.4 ms, TE = 4.5 ms, flip angle = 60°, receiver bandwidth = 219 Hz/pixel, 500 averages per dynamic, 3 dynamics). Data were reconstructed into a 32 × 32 × 7 matrix with a voxel size of 15 × 15 × 25 mm^3^. The sequence, lasting approximately eight minutes, was conducted thrice without a notable interval and the three scan dynamics were averaged in complex form.

### 2.3 Image processing and analysis

**Fig. 1:**
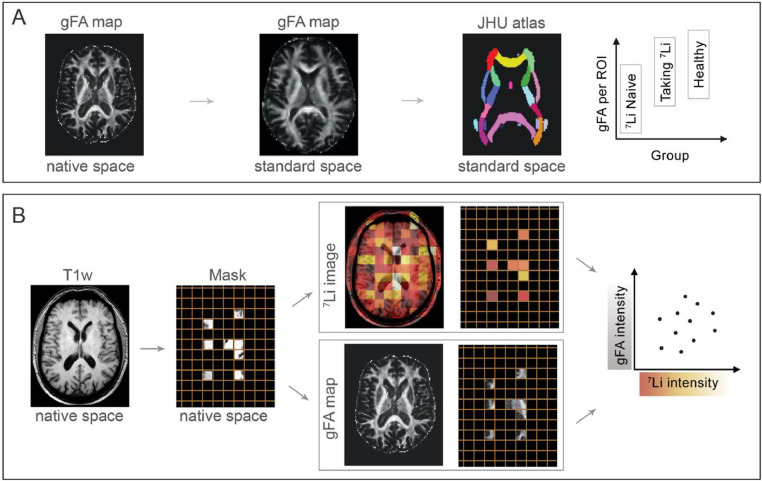
Schematic outline of processing pipeline. (A) gFA ROI analysis between groups was performed by transforming the gFA maps into standard space and applying the John Hopkins University (JHU) atlas. (B) The gFA ∼ lithium analysis for each subject was performed in native space at the resolution of the lithium image. The T1w image was used to identify lithium voxels containing a certain percentage of white matter, which essentially creates a mask. The mask was applied to both the gFA map and the ^7^Li-MRI image. Finally, mean gFA in each mask voxel was plotted against the ^7^Li signal. Hence, each data point corresponds to a lithium voxel in a subject.

All T1w, dMRI and ^7^Li-MRI images were exported in DICOM format and converted to NIFTI format data using the Matlab toolbox ‘DICOM to NIfTI’ (Xiangrui, 2017). Data pre-processing and analysis were performed using Nipype, a Python based platform that provides a uniform interface to existing neuroimaging software and facilitates interaction between these packages within a single workflow (Gorgolewski et al., 2011). An outline of the processing pipeline is shown in Fig. 1.

*T1w image processing:* T1w structural images acquired during the ^1^H imaging session were registered to the standard Montreal Neurological Institute (MNI) 2 mm brain using the Functional MRI of the Brain (FMRIB) Linear and Non Linear Image Registration Tools (Jenkinson et al., 2012). These images were also linearly co-registered to the T1w structural image acquired during the ^7^Li imaging session using FMRIB's Linear Image Registration Tool (Jenkinson et al., 2012). T1w structural images acquired during the ^7^Li imaging session were segmented into three tissue classes (grey matter, white matter and cerebrospinal fluid) using the FMRIB Automated Segmentation Tool (Zhang et al., 2001).

*Diffusion-weighted image processing:* dMRI images from every subject were corrected for motion and eddy-current distortions using the eddy correct and bvec rotation utility in the FMRIB Software Library (FSL) (Andersson and Sotiropoulos, 2016). The b0 image was used as the reference image for realignment of all the dMRI images, and mean displacement for each subject across the acquisition was calculated. One-way ANOVA failed to reveal a significant difference between the mean displacement between the three groups (*p* = 0.64). Subsequently, skull-stripping was performed on the b0 image using FSL's Brain Extraction Tool (Smith et al., 2002) and images were visually inspected to ensure skull-stripping had occurred successfully. A Constant Solid Angle (Q-Ball) model with fourth-order spherical harmonics was fitted to motion-corrected dMRI images using the Python package Dipy (version 0.11) (Garyfallidis et al., 2014) yielding an estimation of gFA for each brain voxel. gFA was chosen in favour of FA owing to the ability of the Q-Ball model to account for crossing fibres (Tuch, 2004). gFA maps were co-registered with T1w structural images using FMRIB's Linear Image Registration Tool (Jenkinson et al., 2012) with six degrees of freedom. gFA maps were transformed into MNI space by applying the transformation matrix obtained from registering the structural T1w images to the standard MNI 2 mm brain using FMRIB's Non Linear Image Registration Tool (Andersson et al., 2007).

For the analysis of mean gFA across the white matter for each subject in MNI space, the mean gFA across all voxels in the John Hopkins University (JHU) White-Matter Tractography ROI atlas (Wakana et al., 2004) was calculated. To quantify differences across groups (BDC, BDL, and HC), the effects of age and sex were regressed out and the residuals were compared between the groups. One-way ANOVA was used to test for group differences in mean white matter gFA residuals, followed by post-hoc t-tests (applying Tukey's correction for multiple comparisons) to determine the direction of effect. The statistical significance threshold was set at *p* < 0.05. Fig. 1A shows a schematic outline of this processing step.

For the ROI based analysis of gFA in MNI space, region-wise differences in mean gFA were determined for each JHU ROI by computing effect sizes (Cohen's *d*) between the BDL and BDC groups. Effect sizes were computed using residuals after regressing out the effects of age and sex for each individual ROI. Estimating the statistical significance of ROI-specific changes was avoided because of the small sample sizes, with the measured effect sizes serving to demonstrate spatial heterogeneity of the effects between groups. In other words, this analysis did not seek to determine whether any ROIs differed significantly in gFA between groups, but more cautiously explored whether there was spatial heterogeneity in the effects between groups.

*^7^Li-MRI image processing:* ^7^Li-MRI image analysis was performed in native space due to the relatively low spatial resolution of these images (voxel size 24.0 × 25.3 × 25.0 mm^3^). After segmentation of the T1w structural image in subject native space, percentage tissue content (grey matter, white matter and cerebrospinal fluid) was estimated for each ^7^Li-MRI voxel. gFA maps were linearly registered to the same subject space and the relationship between mean gFA and lithium signal within each ^7^Li-MRI voxel was investigated at white matter thresholds ranging from 0 to 100% in steps of 25%. In essence, we performed all analyses steps at the resolution of the lithium images in subject space. This ensured that no effect of up-sampling, or interpolation of low resolution images, would affect our results. Fig. 1B shows a schematic outline of this processing step.

To enable a group analysis of the association between gFA and ^7^Li-MRI signal intensity, a linear mixed effects model was used to account for inter-subject variations (Winter, 2013). As fixed effects, we entered gFA, age and sex (without interaction term) into the model. As random effects, we had intercepts for subjects and by-subject random slopes for the effect of ^7^Li-MRI signal. Visual inspection of residual plots did not reveal any obvious deviations from homoscedasticity or normality. P values were obtained by likelihood ratio tests of the full model with the fixed effect of ^7^Li-MRI signal against the model without the fixed effect of ^7^Li-MRI signal assuming a significance threshold of *p* < 0.05. Supplementary Information A shows the full linear mixed effect analysis and additional tests to determine the random effects.

*Supplementary analysis:* The results presented in the body of the manuscript are derived from the gFA analysis. To facilitate comparison of our data with other studies in bipolar disorder, a standard Fractional Anisotropy analysis was also performed (including the determination of the spatial relationship with ^7^Li-MRI signal intensity). The analysis pipeline was identical to that described for gFA, except that the FSL ‘dtifit’ function was used to generate the FA maps. The process and results of this analysis are provided in Supplementary Information B, qualitatively matching the gFA findings below, with only minor differences in effect size.

## 3 Results

### 3.1 Group characteristics

Twelve BDL subjects (five women; mean age: 46 ± 13 SD years), seventeen BDC subjects (eleven women; mean age: 44 ± 12 SD years) and sixteen HC subjects (ten women; mean age: 49 ± 3 SD years) were included in the analysis. One BDL subject was excluded from the ^7^Li-MRI analysis because their lithium imaging data was acquired during the ^7^Li-MRI sequence development phase and the acquisition protocol was inconsistent with the other subjects in the study. The groups did not differ in mean age (*F* = 0.6; *p* = 0.77) or sex distribution (χ^2^ = 1.8; *p* = 0.45) but there was a difference in duration of education (*F* = 5.3; *p* = 0.01) (Table 1). Post-hoc testing revealed that the HC group remained in education for longer than the BDL group (*t* = 2.59; *p* = 0.03) and the BDC group (*t* = 2.95; *p* = 0.01), but the bipolar disorder groups did not differ in years of education (*t* = 0.12; *p* = 0.9). Regarding illness characteristics, the BDL and BDC groups did not differ in duration of illness (Mann–Whitney *U* = 79.0; *p* = 0.66), subtype (BD I versus II, Fisher's exact test *p* = 0.11), history of psychosis (χ^2^ = 0.36; *p* = 0.55) or presence of co-morbid psychiatric diagnosis (Fisher's exact test *p* = 0.42). The groups differed in HAM-D scores (*F* = 3.97; *p* = 0.03), the HC group having lower scores than the BDC group (*t* = 2.65; *p* = 0.03) but not the BDL group (*t* = 2.09; *p* = 0.10); the BDL and BDC groups did not differ in depression rating scores (*t* = −0.33; *p* = 0.94) and all were euthymic.

**Table 1:** Subject characteristics.

	Bipolar disorder Lithium (*n* = 12)	Bipolar disorder control (*n* = 17)	Healthy Control (*n* = 16)	Significance
Sex (M/F)	7/5	6/11	6/10	*p* = 0.45
Age (years)	46(13)	44(12)	49(3)	*p* = 0.77
Educational level (years)	14(2)	14(2)	17(3)	*p* = 0.87
YMRS score	1(1)	1(1)	0.2(0.5)	*p* = 0.96
HAM-D score	5(6)	5(5)	1(1)	*p* = 0.03
Bipolar disorder subtype (I/II)	6/6	3/14	n/a	*p* = 0.11
History of psychosis (present/absent)	5/7	9/8	n/a	*p* = 0.55
Secondary diagnosis present	58%	76%	n/a	*p* = 0.42
Duration of illness (years)	9.5(9.9)	10.46(8.9)	n/a	*p* = 0.66
Duration of lithium treatment (years)	8(9)	n/a	n/a	n/a
Priadel™ dose (mg)	900(130)	n/a	n/a	n/a
Serum lithium concentration (mmol/L)	0.8(0.13)	n/a	n/a	n/a
LISERS score	17(13)	n/a	n/a	n/a

YMRS: Young Mania Rating Scale. HAM-D: Hamilton Rating Scale for Depression. LISERS: Lithium Side Effects Rating Scale. Values reported as mean (standard deviation).

All subjects with bipolar disorder were taking some form of medication at time of scanning. Fisher's exact tests were performed in order to compare the use of medication by class between diagnostic groups. Barring lithium, no significant differences were found between the bipolar disorder groups across all major medication classes. Medication usage information for each group and their corresponding *p* values are provided in Table 2.

**Table 2:** Medication use by class. Fisher's exact tests found no differences in medication class between diagnostic groups other than lithium (OR: adds ratio).

Medication class	Bipolar disorder lithium (*n* = 12)	Bipolar disorder controls (*n* = 17)	Significance
Antipsychotics	8 (66%)	11 (65%)	OR = 1.1, *p* = 1
Antidepressants	6 (50%)	10 (59%)	OR = 0.7, *p* = 0.7
Anticonvulsants	5 (42%)	10 (59%)	OR = 0.5, *p* = 0.5
Anxiolytics	3 (25%)	4 (24%)	OR = 1.1, *p* = 1
Hypnotic	3 (25%)	2 (12%)	OR = 2.5, *p* = 0.6
Antihistamine	0	1 (6%)	OR = 0, *p* = 1
Over the counter	0	1 (6%)	OR = 0, *p* = 1
Mood stabilisers/mania	12 (lithium, 100%)	0	n/a

### 3.2 Mean white matter gFA comparison between groups

**Fig. 2:**
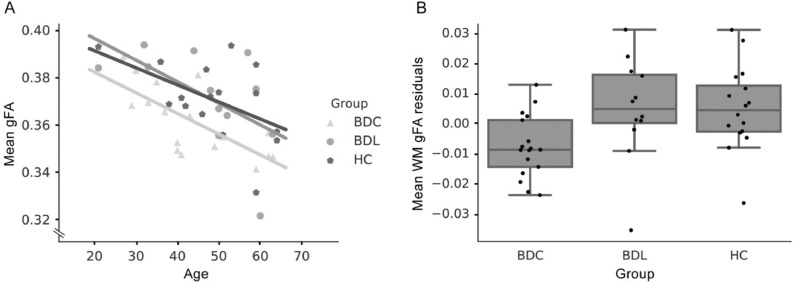
gFA group comparison. (A) Association between mean white matter gFA and age. Each dot is an individual subject and lines indicate the least squared regressions. (B) Group comparison of mean gFA residuals after correction for age and sex.

A significant negative correlation (*r* = −0.52; *p* = 0.0002) between mean white matter gFA and age was found across all subjects. This was anticipated as FA has previously been shown to be negatively associated with age in adulthood (Lebel et al., 2010). After regressing out the effects of age and sex, ANOVA revealed a significant difference between the groups (*F* = 4.05; *p* = 0.02; Fig. 2B) and post hoc tests revealed that the BDL and HC groups exhibited higher mean white matter gFA residuals compared to the BDC group (BDL vs BDC, *t* = 2.5; *p* = 0.05)(HC vs BDC, *t* = 2.7; *p* = 0.03). No differences were found between the BDL and HC groups (*t* = 0.02 *p* = 1).

### 3.3 Region of interest gFA group comparison

**Fig. 3:**
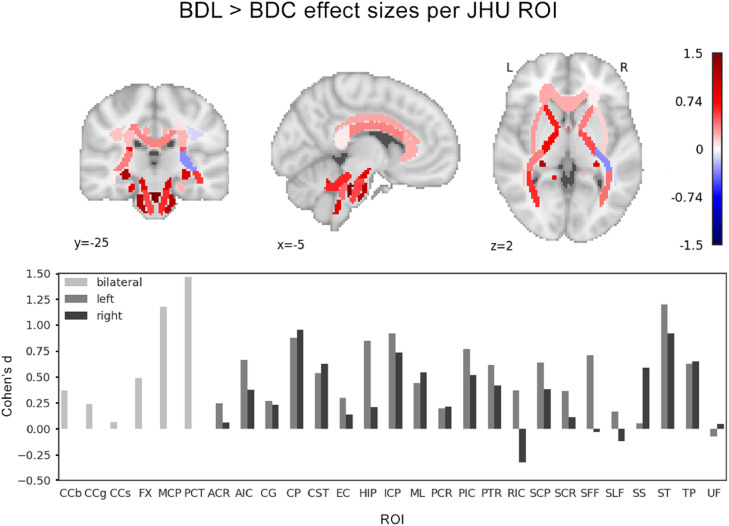
Region-wise gFA effect sizes (Cohen's *d*) per JHU ROI comparing BDL > BDC. Top: JHU ROIs in standard space are colour coded according to the effect size of BDL > BDC. Bottom: Bar plot showing individual effect sizes for each ROI. Full ROI labels and all effect sizes are provided in Supplementary A. Note effect sizes are calculated on residual gFA values after age and sex correction for each ROI. (For interpretation of the references to colour in this figure legend, the reader is referred to the web version of this article.)

Region-wise gFA effect sizes for the comparison BDL > BDC are shown in Fig. 3. Subjects taking lithium exhibited higher gFA in 44 out of 48 ROIs compared to those taking other medications for bipolar disorder but naïve to lithium, with eight regions exhibiting an effect size of greater than 0.8 (signifying a large effect). Effect sizes ranged from −0.3 to +1.5, indicating spatial heterogeneity in gFA differences between the groups. No significant difference was observed in the magnitude of the effects sizes when comparing the ROIs in the right and left hemispheres (*p* = 0.19 in a rank-sum test). All ROI labels and their corresponding effect sizes are provided in Supplementary Information A.

### 3.4 Co-localisation of lithium and gFA

**Fig. 4:**
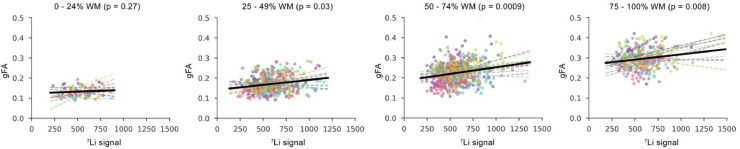
Relationship between lithium signal amplitude and gFA. Relationship between ^7^Li-MRI signal intensity and mean gFA in ^7^Li-MRI voxels containing varying levels of white matter (ranging from 0 to 100% in steps of 25%). Colours represent individual subject data points and dashed lines represent individual subject least-squares regression line. The black line represents the group linear mixed effects regression line. *p* values represent significance of a theoretical likelihood test comparing linear mixed effect models which do and do not include ^7^Li signal as a fixed effect. (For interpretation of the reference to colour in this figure legend, the reader is referred to the web version of this article.)

The relationship between white matter integrity and the spatial distribution of lithium was determined in a linear mixed effect analysis of ^7^Li-MRI signal intensity and gFA values in voxels containing varying proportions of white matter at the resolution of the ^7^Li-MRI scan (Fig. 4). Results revealed a highly significant association between the ^7^Li-MRI signal and gFA (*p* < 0.01) once white matter content exceeded 50% in the ^7^Li-MRI voxel of interest.

Linear mixed effect modelling was used to test a random slope model against a random intercept model to determine whether there was evidence to suggest that individual subjects need to be modelled with individual slopes. These results revealed there was a significant difference in the models accounting for a subject specific slope versus one that does not (*p* < 0.05). The full linear mixed effect analysis is described in detail in Supplementary Information A.

## 4 Discussion

In this study, we report that patients with bipolar disorder treated with lithium have white matter gFA values comparable to healthy controls and greater than bipolar disorder patients receiving other medications. Further, as hypothesised, the spatial distribution of the ^7^Li-MRI signal positively correlated with gFA in those prescribed lithium.

There is a growing literature suggesting that in bipolar disorder, lithium treatment positively influences white matter integrity, as determined using diffusion MRI techniques (Hafeman et al., 2012; Kafantaris et al., 2017). We sought to determine whether there was a spatial association between gFA and the distribution of lithium in the brain, given our recent observation of greater ^7^Li-MRI signal intensity in white matter compared to grey matter (Smith et al., 2018). Whilst dMRI techniques are well established, our combined analysis of gFA and ^7^Li-MRI data was novel and so exploratory in nature. A major challenge in this analysis was that the lithium images were relatively low resolution compared to the T1w and dMRI images, meaning that a single ^7^Li-MRI voxel often contained mixed tissue types. To enable a fair comparison, all analyses using the ^7^Li-MRI data were performed in native space at the resolution of the lithium image. Results from our linear mixed effect model show the emergence of a robust and highly significant effect of the ^7^Li-MRI signal in explaining gFA in ^7^Li-MRI image voxels comprised of 50% white matter or more. Further, we found a significant difference between the random intercept model and the random slope model, potentially suggesting subject-specific slopes of the gFA ∼ lithium relationship. However, these results attained statistical power through the use of linear mixed effect modelling by estimating associations on the BDL group as a whole. Our data did not permit us to derive reliable subject specific slopes. Hence, at the present stage, we cannot conclude the significance of individual slopes, nor can we infer the causality of the association between gFA and lithium. However, future work with a larger sample will determine whether individual subject-specific slopes are a robust finding and if so, explore their relationship to serum lithium concentrations and clinical indices such as response to lithium, side-effect profiles and cognitive function.

Lithium is purported to exert a range of neurorestorative effects, many of which could influence white matter microstructure and affect gFA. For instance, lithium is known to inhibit glycogen synthase kinase 3β and inhibition of this enzyme increases the number of oligodendrocytes and promotes peripheral nerve myelination in mice (Makoukji et al., 2012; Azim and Butt, 2011). Lithium treatment has been shown to be positively associated with MRI measures of white matter integrity (Gildengers et al., 2015; Haarman et al., 2016; Macritchie et al., 2009) as well as protect against a decrease in white matter volume over time (Berk et al., 2017). Lithium was associated with an increase in FA in the cingulum hippocampus in a recent longitudinal study of adolescents with bipolar disorder, where responders to treatment showed the greatest percentage increase in FA (Kafantaris et al., 2017). However, the lack of a treatment comparator group limits the extent to which the FA change can be specifically attributed to lithium.

Lithium-related alterations in water homeostasis also require consideration in the interpretation of its effects on dMRI-derived measures. Rats fed lithium for five weeks have been shown to have greater brain tissue water content compared to placebo controls (Phatak et al., 2006) and it has been argued that this may underpin the volumetric changes detected in neuroimaging studies in bipolar disorder (Regenold, 2008). However, the differences in water content reached significance in the frontal cortex only, suggesting regional specificity of action but detracting from the explanation that water content change accounts for the more widespread cortical differences in grey matter volume on MRI. Increased water content driven by lithium cannot easily explain its effects on FA either, as widespread increases in free water content within white matter has been linked to decreased FA, at least in first-episode patients with psychosis (Lyall et al., 2018). Notably in the current study, the bipolar groups did not differ in the presence or absence of a history of psychosis during mania or depression.

In practice, lithium may be preferentially prescribed to those deemed more likely to respond to it, to those with more severe presentations or when there is confidence that the patient will manage regular blood level monitoring. It could be argued that the differences in FA between patients treated with lithium and those on other medications reflects these, or other as yet unidentified illness characteristics driving a selection bias rather than a direct action of lithium. For instance, patients with more severe illnesses (BD I) are known to have lower white matter FA values compared to those with milder presentations (BD II) (Foley et al., 2018). If our lithium treated group were composed mostly of patients with a mild illness form, illness severity rather than the effects of lithium might account for our gFA findings. We found no significant difference in the distribution of disorder subtypes in or bipolar groups. Numerically but not significantly, BD I outweighed BD II in the lithium treated group – one might therefore have expected the FA values in the BDL group to be lower on the basis of illness severity but they did not differ from the healthy controls. Our finding of a relationship between gFA and regional lithium concentration strengthens the argument that lithium directly influences white matter integrity.

*Future work:* In future work, multimodal imaging studies utilising ^7^Li-MRI and larger cohorts that permit the segregation of subjects into groups based upon lithium response may reveal localised response-specific relationships between regional brain lithium concentration and MRI derived measures such as gFA. Randomised controlled longitudinal studies would be required to properly assess the relationship between lithium treatment, gFA change and response, and such investigations would be strengthened by inclusion of ^7^Li-MRI examination. Ultimately, such approaches may help characterise lithium responders and move us a step closer to personalised medicine in bipolar disorder. The development of multimodal ^7^Li-MRI analysis techniques affords the opportunity to better investigate the effect of lithium *in vivo*, and we can envisage studies that may advance our understanding of the mode of action of this important mood stabilising drug. The combination of ^7^Li-MRI and proton magnetic resonance spectroscopic imaging (^1^H-MRSI), for instance, could be used to explore the dose-related effects of lithium on the neurometabolites implicated in its actions. The comparable resolution of ^7^Li-MRI and ^1^H-MRSI lends itself to this combination.

*Limitations:* Given the exploratory nature of our analysis using a relatively small cohort of patients, our results should be interpreted with caution. Whilst we found a significantly greater mean white matter gFA in long-term lithium-treated patients relative to lithium naïve patients, we cannot infer regional specificity. The detection of statistically significant region-specific changes requires larger cohorts factoring in corrections for multiple comparisons or synthesis by meta-analysis (Wise et al., 2016; Pezzoli et al., 2018). Further, the analysis exploring the relationship between gFA and ^7^Li-MRI signal intensity was conducted in native space and as a result, we are not able to comment on the anatomical location of voxels by white matter quartile for the group. In our cross sectional study, we recruited patients for whom lithium had been considered clinically by their treating team and our groups comprised a mixture of bipolar disorder type I and II. Whilst the subtype proportion did not differ between the groups, future studies may benefit from the investigation of purer samples.

In conclusion, we report dMRI results that are consistent with the previous literature and a novel observation of the correlation between gFA and regional brain lithium concentration. ^7^Li-MRI is a new technique that holds potential to provide multiple insights into the effects of lithium in the brain in bipolar disorder. However, the limited spatial resolution of this technique restricted the present study to reporting results based on voxels containing mixed tissue types. Despite this, we found a consistent significant relationship between ^7^Li-MRI signal and gFA in voxels containing 50% white matter or more. Whilst challenging, there is scope for on-going technical development of ^7^Li-MRI with clear potential to improve the spatial resolution of lithium images. The ability to co-localise a psychotropic medication with its effect *in vivo* spurs us to meet these challenges.

## 5 Disclosures

The authors declare no conflict of interest and the funders played no role in the design or analysis of the study.

## Appendix Supplementary materials

Image, application 1

Image, application 2

Image, application 3
