# Supplementary material for: White matter microstructural properties in bipolar disorder in relationship to the spatial distribution of lithium in the brain
Source: J Affect Disord. 2019 Jun 15;253:224–31. doi: 10.1016/j.jad.2019.04.075 (PMC6609924; doi:10.1016/j.jad.2019.04.075)
Supplement: Supplementary file 1 [file mmc1.docx]

# **SUPPLEMENTARY A:** **Linear mixed effect modelling of the relationship between gFA and ^7^Li-MRI signal**

*75-100% white matter (WM) linear mixed effect analysis*

To investigate the association of the ^7^Li-MRI signal (Li) with gFA, while accounting for the inter-subject variation, we tested the two following random slope models against each other:

lme_rs: gFA ~ age + sex +(1+Li|subject)

lmealt_rs: gFA ~ Li + age + sex +(1+Li|subject)

The output was:

*Theoretical Likelihood Ratio Test*

| Model | DF | AIC | BIC | LogLik | LRStat | deltaDF | *p*-value |
| --- | --- | --- | --- | --- | --- | --- | --- |
| lme_rs | 7 | -982.47 | -956.43 | 498.23 |  |  |  |
| lmealt_rs | 8 | -987.43 | -957.66 | 501.71 | 6.9561 | 1 | 0.0083536 |

We concluded that the ^7^Li-MRI signal shows a significant effect on gFA.

We also tested the random slope model above against a random intercept model:

lmealt_rs: gFA ~ Li + age + sex +(1+Li|subject)

lmealt_ri: gFA ~ Li + age + sex +(1|subject)

*Theoretical Likelihood Ratio Test*

| Model | DF | AIC | BIC | LogLik | LRStat | deltaDF | *p*-value |
| --- | --- | --- | --- | --- | --- | --- | --- |
| lmealt_ri | 6 | -983.35 | -961.03 | 497.68 |  |  |  |
| lmealt_rs | 8 | -987.43 | -957.66 | 501.71 | 8.0719 | 2 | 0.017669 |

We conclude from this that the random effect affects the intercept and slope, and that there is evidence to suggest a subject specific slope.

**Linear mixed effect analysis for other ^7^Li-MRI voxel WM percentages**

The *p*-values reported in the main paper (Figure 4) are based on the comparison of lme_rs versus lmealt_rs:

| WM % | *p*-value of lme_rs vs. lmealt_rs | Slope estimate |
| --- | --- | --- |
| 75-100% | 0.0083536 | 5.8411e-05 (2.1665e-05; 9.5157e-05) |
| 50-74% | 0.00090728 | 5.715e-05 (3.2175e-05; 8.2125e-05) |
| 25-49% | 0.028726 | 4.0996e-05 (1.036e-05; 7.1633e-05) |
| 0-24% | 0.27183 | 2.2251e-05 (-1.6315e-05; 6.0817e-05) |

For the comparison of the random slope versus random intercept model we found a difference at 50-74% WM and 25-49% WM (with *p* = 0.05 and *p* = 0.04 respectively). However, we did not find a significant difference (*p* = 0.96) at 0-24% WM.

Overall, our linear mixed effect analysis suggests that at least for regions with at least 50% WM, the ^7^Li-MRI signal is a significant predictor of gFA (*p* < 0.01). However, the exact predictive relationship (in terms of both slope and offset of a regression) may be subject specific.

# **JHU ROI labels names and gFA effect sizes for BDL > BDC**

| ROI name | ROI label | Hemisphere | Effect size (Cohen’s *d*) |
| --- | --- | --- | --- |
| Anterior corona radiata | ACR | left | 0.246210523 |
| Anterior corona radiata | ACR | right | 0.057806622 |
| Anterior limb of internal capsule | AIC | left | 0.662554627 |
| Anterior limb of internal capsule | AIC | right | 0.373515902 |
| Body of corpus callosum | CCb | bilateral | 0.367416635 |
| Genu of corpus callosum | CCg | bilateral | 0.235718001 |
| Splenium of corpus callosum | CCs | bilateral | 0.065361703 |
| Cingulate gyrus | CG | left | 0.265657272 |
| Cingulate gyrus | CG | right | 0.233199424 |
| Cerebral peduncle | CP | left | 0.875567924 |
| Cerebral peduncle | CP | right | 0.952483946 |
| Corticospinal tract | CST | left | 0.533640802 |
| Corticospinal tract | CST | right | 0.625839718 |
| External capsule | EC | left | 0.295155738 |
| External capsule | EC | right | 0.133147153 |
| Fornix | FX | bilateral | 0.490255966 |
| Hippocampus | HIP | left | 0.848861444 |
| Hippocampus | HIP | right | 0.209719588 |
| Inferior cerebellar peduncle | ICP | left | 0.917543474 |
| Inferior cerebellar peduncle | ICP | right | 0.733652766 |
| Middle Cerebellar Peduncle | MCP | bilateral | 1.173611802 |
| Medial lemniscus | ML | left | 0.438665543 |
| Medial lemniscus | ML | right | 0.540423021 |
| Posterior corona radiata | PCR | left | 0.197073392 |
| Posterior corona radiata | PCR | right | 0.215035233 |
| Pontine Crossing Tract | PCT | bilateral | 1.48461202 |
| Posterior limb of internal capsule | PIC | left | 0.769347052 |
| Posterior limb of internal capsule | PIC | right | 0.520571668 |
| Posterior thalamic radiation | PTR | left | 0.611596166 |
| Posterior thalamic radiation | PTR | right | 0.41694824 |
| Retrolenticular part of internal capsule | RIC | left | 0.371712476 |
| Retrolenticular part of internal capsule | RIC | right | -0.324872563 |
| Superior cerebellar peduncle | SCP | left | 0.639062405 |
| Superior cerebellar peduncle | SCP | right | 0.382813258 |
| Superior corona radiata | SCR | left | 0.362389352 |
| Superior corona radiata | SCR | right | 0.113772052 |
| Superior fronto-occipital fasciculus | SFF | left | 0.709596925 |
| Superior fronto-occipital fasciculus | SFF | right | -0.032606845 |
| Superior longitudinal fasciculus | SLF | left | 0.166852087 |
| Superior longitudinal fasciculus | SLF | right | -0.120176496 |
| Sagittal stratum | SS | left | 0.050430933 |
| Sagittal stratum | SS | right | 0.589158978 |
| Stria terminalis | ST | left | 1.197598757 |
| Stria terminalis | ST | right | 0.91909396 |
| Tapetum | TP | left | 0.627411394 |
| Tapetum | TP | right | 0.650012872 |
| Uncinate fasciculus | UF | left | -0.070487198 |
| Uncinate fasciculus | UF | right | 0.048170358 |
