# Supplementary material for: White matter microstructural properties in bipolar disorder in relationship to the spatial distribution of lithium in the brain
Source: J Affect Disord. 2019 Jun 15;253:224–31. doi: 10.1016/j.jad.2019.04.075 (PMC6609924; doi:10.1016/j.jad.2019.04.075)
Supplement: Supplementary file 2 [file mmc2.docx]

# **SUPPLEMENTARY INFORMATION B: AN INVESTIGATION INTO THE RELATIONSHIP BETWEEN LITHIUM AND FRACTIONAL ANISOTROPY**

**Summary of FA analysis results**

The same analysis pipeline as used during the gFA analysis was used to determine the relationship between FA and lithium. All figures included in the main manuscript have been reproduced using FA (derived from FSL ‘dtifit’ function) instead of gFA. In short, all results and trends were found to be qualitatively the same, namely, higher FA in patients taking lithium, and a positive association between ^7^Lithium signal and FA magnitude.

The main differences between the gFA and FA analysis were observed in terms of the magnitude of effect sizes (see Figure 2). The maximal effect size fell from 1.48 to 1.19. and fewer regions (41 vs 44) exhibited large (d > 0.8) effect sizes. Notably, the maximal effect size discrepancy between FA and gFA was observed in the Pontine Crossing Tract, an area of high fibre crossing. We hypothesise that this difference in effect size reflects the ability of gFA to act as a better measure of anisotropy in such areas containing crossed fibres. Results for the FA analysis are provided below.

**Mean white matter FA comparison between groups**

A significant negative correlation (r = -0.59; *p* = 0.0002) between mean white matter FA and age was found across all subjects. After regressing out the effects of age and sex, ANOVA revealed a trend difference between the groups (F(2, 42) = 3.05; *p* = 0.035) and post hoc tests revealed that the BDL and HC group exhibited higher mean white matter gFA residuals compared to the BDC group (t = 2.4, *p* = 0.05). The difference between the BDL and BDC group was close to significance (t = 2.2, *p* = 0.09). No difference was found between the BDL and HC groups (t = 0.07, *p* = 1). These results are shown below in Figure 1.


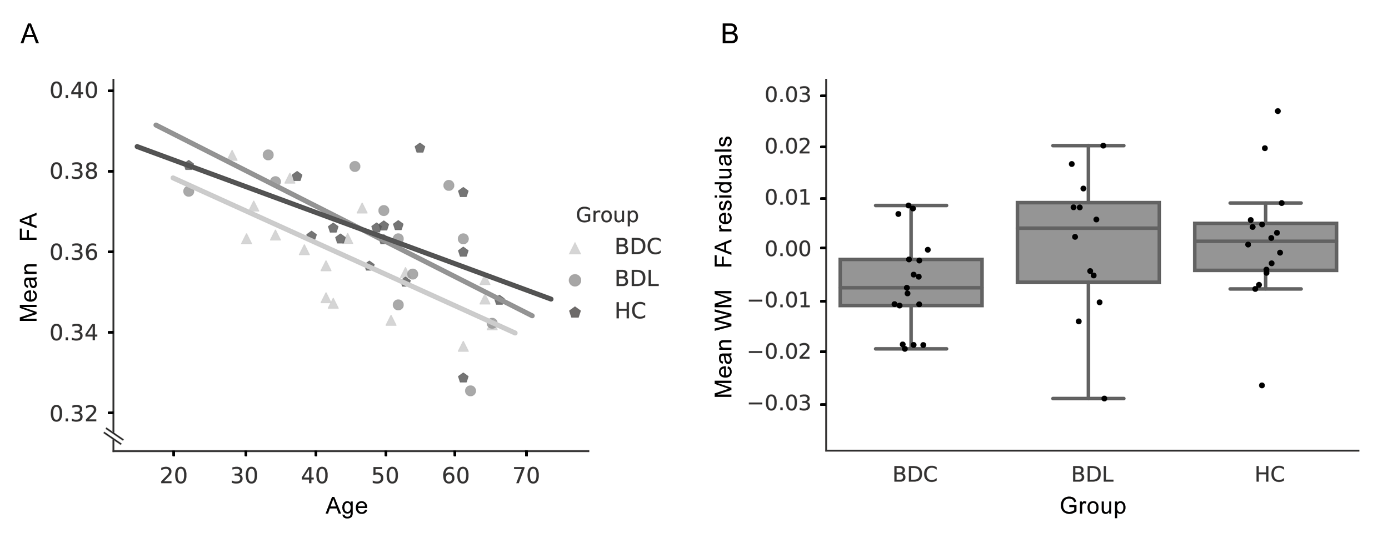


**Figure 1. FA group comparison.** (A) Association between mean white matter FA and age. Each dot is an individual subject and lines indicate the least squared regressions. (B) Group comparison of mean FA residuals after correction for age and sex.

**Region of interest FA group comparison**

Region-wise FA effect sizes for the comparison BDL > BDC are shown below in Figure 2. Subjects taking lithium exhibited higher gFA in 41 out of 48 ROIs compared to those taking other medications for bipolar disorder but naïve to lithium, with three regions exhibiting an effect size of greater than 0.8 (signifying a large effect). Effect sizes ranged from -0.4 to + 1.18, indicating spatial heterogeneity in FA differences between the groups. No significant difference was observed in the magnitude of the effects sizes when comparing the ROIs in the right and left hemispheres (*p* = 0.21 in a ranksum test). All ROI labels and their corresponding effect sizes are provided below in Table 1.


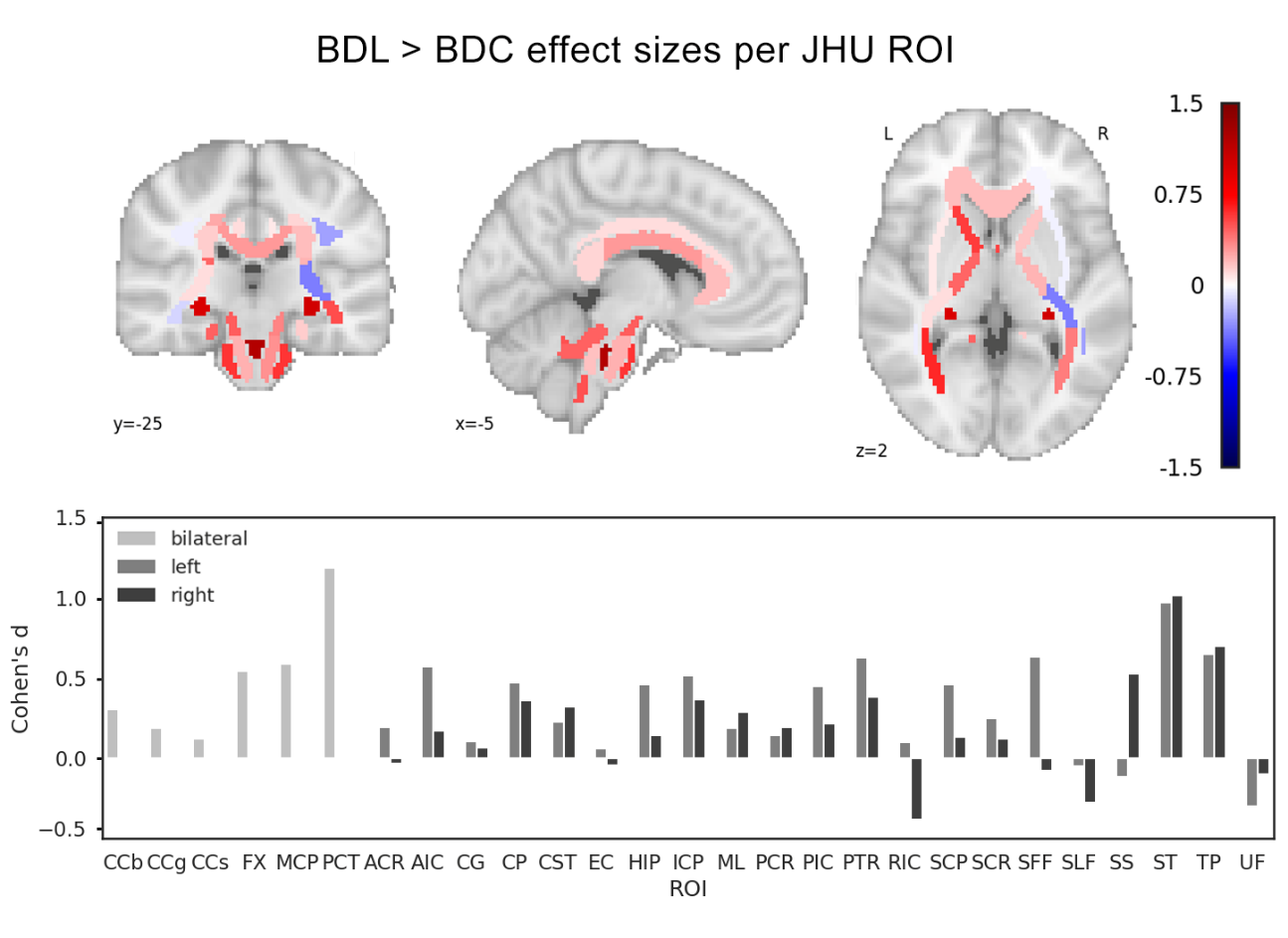


**Figure 2**. **Region-wise FA effect sizes (Cohen’s *d*) per JHU ROI comparing BDL>BDC**. Top: JHU ROIs in standard space are colour coded according to the effect size of BDL>BDC. Bottom: Bar plot showing individual effect sizes for each ROI. Full ROI labels and all effect sizes are provided in Supplementary B. Note effect sizes are calculated on residual FA values after age and sex correction for each ROI.

**Co-localisation of lithium and FA**

The relationship between white matter integrity and the spatial distribution of lithium was determined in a linear mixed effect analysis of ^7^Li-MRI signal intensity and FA values in voxels containing varying proportions of white matter at the resolution of the ^7^Li-MRI scan (Figure 3). Results revealed a highly significant association between the ^7^Li-MRI signal and FA (*p* < 0.01) once white matter content exceeded 50% in the ^7^Li-MRI voxel of interest.

Using linear mixed effect modelling, we also tested the random slope model against a random intercept model to determine whether there was evidence to suggest that individual subjects need to be modelled with individual slopes. These results revealed there was a significant difference in the models accounting for a subject specific slope versus one that does not (*p* < 0.05). The full linear mixed effect analysis is described in detail below.


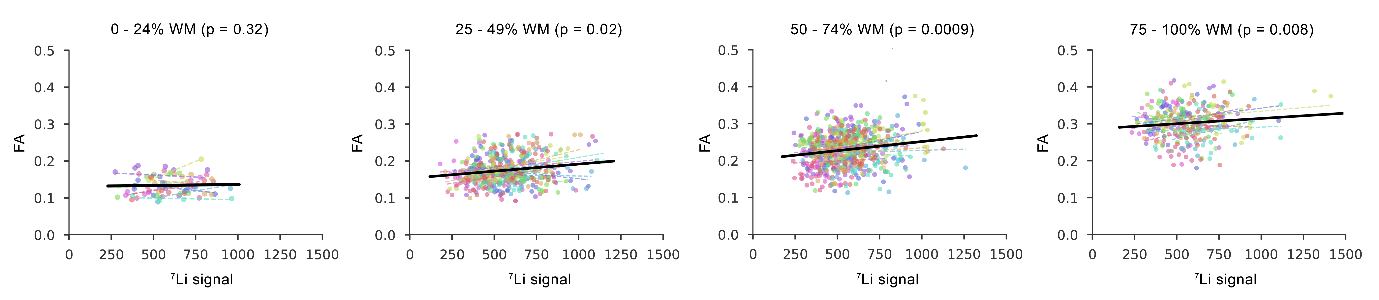


**Figure 3. Relationship between lithium signal amplitude and FA.** Relationship between ^7^Li-MRI signal intensity and mean FA in ^7^Li-MRI voxels containing varying levels of white matter (ranging from 0-100% in steps of 25%). Dashed lines and associated colours represent individual subject­ least-squares regression line. Black line represents group linear mixed effects regression line. *P*-values represent significance of a theoretical likelihood test comparing linear mixed effect models which do and do not include ^7^Li signal as a fixed effect.

# **Linear mixed effect modelling of the relationship between FA and ^7^Li-MRI signal**

*75-100% white matter (WM) linear mixed effect analysis*

To investigate the association of the ^7^Li-MRI signal (Li) with FA, while accounting for the inter-subject variation, we tested the two following random slope models against each other:

lme_rs: FA ~ age + sex +(1+Li|subject)

lmealt_rs: FA ~ Li + age + sex +(1+Li|subject)

The output was:

*Theoretical Likelihood Ratio Test*

| Model | DF | AIC | BIC | LogLik | LRStat | deltaDF | *p*-value |
| --- | --- | --- | --- | --- | --- | --- | --- |
| lme_rs | 7 | -982.469 | -956.42 | 498.23 |  |  |  |
| lmealt_rs | 8 | -987.447 | -957.67 | 501.72 | 6.971 | 1 | 0.008279 |

We concluded that the ^7^Li-MRI signal shows a significant effect on FA.

We also tested the random slope model above against a random intercept model:

lmealt_rs: FA ~ Li + age + sex +(1+Li|subject)

lmealt_ri: FA ~ Li + age + sex +(1|subject)

*Theoretical Likelihood Ratio Test*

| Model | DF | AIC | BIC | LogLik | LRStat | deltaDF | *p*-value |
| --- | --- | --- | --- | --- | --- | --- | --- |
| lmealt_ri | 5 | -974.74 | -956.14 | 492.37 |  |  |  |
| lmealt_rs | 7 | -982.46 | -956.42 | 498.23 | 11.725 | 2 | 0.0028440 |

We conclude from this that the random effect affects the intercept and slope, and that there is evidence to suggest a subject specific slope.

**Linear mixed effect analysis for other ^7^Li-MRI voxel WM percentages**

The p-values reported in the main paper (Figure 4) are based on the comparison of lme_rs versus lmealt_rs:

| WM % | *p*-value of lme_rs vs. lmealt_rs | Slope estimate |
| --- | --- | --- |
| 75-100% | 0.008279 | 5.866e-05 (2.259e-05; 9.473e-05) |
| 50-74% | 0.000907315 | 5.782e-05 (3.291e-05; 8.273e-05) |
| 25-49% | 0.018456 | 4.348e-05 (1.367e-05; 7.323e-05) |
| 0-24% | 0.3172 | 1.986e-05 (-1.832e-05; 5.805e-05) |

For the comparison of the random slope versus random intercept model we found a difference at 50-74% WM and 25-49% WM (with *p* < 0.0001). However, we did not find a significant difference (*p* = 0.93) at 0-24% WM.

Overall, our linear mixed effect analysis suggests that at least for regions with at least 50% WM, the ^7^Li-MRI signal is a significant predictor of FA (*p* < 0.01). However, the exact predictive relationship (in terms of both slope and offset of a regression) may be subject specific.

**JHU ROI labels names and FA effect sizes for BDL > BDC**

| ROI name | ROI label | Hemisphere | Effect size (Cohen’s *d*) |
| --- | --- | --- | --- |
| Anterior corona radiata | ACR | left | 0.192381865 |
| Anterior corona radiata | ACR | right | -0.035062586 |
| Anterior limb of internal capsule | AIC | left | 0.569310087 |
| Anterior limb of internal capsule | AIC | right | 0.169049245 |
| Body of corpus callosum | CCb | bilateral | 0.304430838 |
| Genu of corpus callosum | CCg | bilateral | 0.18841078 |
| Splenium of corpus callosum | CCs | bilateral | 0.122048767 |
| Cingulate gyrus | CG | left | 0.104126899 |
| Cingulate gyrus | CG | right | 0.064938126 |
| Cerebral peduncle | CP | left | 0.472513995 |
| Cerebral peduncle | CP | right | 0.362093162 |
| Corticospinal tract | CST | left | 0.229011026 |
| Corticospinal tract | CST | right | 0.320094892 |
| External capsule | EC | left | 0.061587343 |
| External capsule | EC | right | -0.046002306 |
| Fornix | FX | bilateral | 0.542312504 |
| Hippocampus | HIP | left | 0.457141929 |
| Hippocampus | HIP | right | 0.141703698 |
| Inferior cerebellar peduncle | ICP | left | 0.513940044 |
| Inferior cerebellar peduncle | ICP | right | 0.365127359 |
| Middle Cerebellar Peduncle | MCP | bilateral | 0.588535655 |
| Medial lemniscus | ML | left | 0.186198745 |
| Medial lemniscus | ML | right | 0.286170439 |
| Posterior corona radiata | PCR | left | 0.142744474 |
| Posterior corona radiata | PCR | right | 0.195849923 |
| Pontine Crossing Tract | PCT | bilateral | 1.18634506 |
| Posterior limb of internal capsule | PIC | left | 0.44930465 |
| Posterior limb of internal capsule | PIC | right | 0.213309747 |
| Posterior thalamic radiation | PTR | left | 0.623189408 |
| Posterior thalamic radiation | PTR | right | 0.381074613 |
| Retrolenticular part of internal capsule | RIC | left | 0.100097992 |
| Retrolenticular part of internal capsule | RIC | right | -0.382884545 |
| Superior cerebellar peduncle | SCP | left | 0.462003123 |
| Superior cerebellar peduncle | SCP | right | 0.132129321 |
| Superior corona radiata | SCR | left | 0.250498208 |
| Superior corona radiata | SCR | right | 0.120160204 |
| Superior fronto-occipital fasciculus | SFF | left | 0.6327494 |
| Superior fronto-occipital fasciculus | SFF | right | -0.079182174 |
| Superior longitudinal fasciculus | SLF | left | -0.05158399 |
| Superior longitudinal fasciculus | SLF | right | -0.276458334 |
| Sagittal stratum | SS | left | -0.119362047 |
| Sagittal stratum | SS | right | 0.523705248 |
| Stria terminalis | ST | left | 0.970486975 |
| Stria terminalis | ST | right | 1.01144249 |
| Tapetum | TP | left | 0.645564798 |
| Tapetum | TP | right | 0.698649088 |
| Uncinate fasciculus | UF | left | -0.298338392 |
| Uncinate fasciculus | UF | right | -0.099663978 |
